# Metabolomic Response of Tomatoes (*Solanum lycopersicum* L.) against Bacterial Wilt (*Ralstonia solanacearum*) Using ^1^H-NMR Spectroscopy

**DOI:** 10.3390/plants10061143

**Published:** 2021-06-03

**Authors:** Rudi Hari Murti, Enik Nurlaili Afifah, Tri Rini Nuringtyas

**Affiliations:** 1 Department of Agronomy, Faculty of Agriculture, Universitas Gadjah Mada, Yogyakarta 55281, Indonesia; eniknurlaili21@ugm.ac.id; 2 Faculty of Biology, Universitas Gadjah Mada, Yogyakarta 55281, Indonesia; tririni@ugm.ac.id

**Keywords:** bacterial wilt, defense mechanism, leucine, metabolites, nuclear magnetic resonance, tomato, valine

## Abstract

*Ralstonia solanacearum* is the pathogen responsible for wilting, yield losses, and death in tomato plants. The use of resistant cultivars has been proven as the most appropriate solution to controlling this pathogen. Therefore, further study of host-plant resistance mechanisms in tomatoes is urgently needed. ^1^H-NMR (nuclear magnetic resonance) spectroscopy combined with multivariate data analysis has been used to identify the biochemical compounds that play a crucial role in the defense mechanisms of tomato against bacterial wilt. Eleven metabolites consisting of amino acids, sugars and organic acids were identified and presented at different concentrations in each cultivar. Leucine and valine were determined as distinguishable metabolites of resistant and susceptible cultivars. Permata and Hawaii 7996 as resistant cultivars had a significant decrease of valine after inoculation about 1.5–2 times compared to the susceptible cultivar (GM2). Meanwhile, the resistant cultivars had a higher level of leucine, about 1.3–1.5 times compared to the susceptible ones. Synthesis of leucine and valine are linked as a member of the pyruvate family. Therefore, the decrease in valine may be related to the higher need for leucine to form the leucine-rich receptor, which plays a role in the plant’s immune system against the bacterial wilt.

## 1. Introduction

Bacterial wilt caused by the soil-borne pathogen *Ralstonia solanacearum* is responsible for the greatest economic losses in tomato production [1]. This bacterium has a wide host plants and causes severe yield losses in many crops, including tomato, eggplants, tobacco, potato, and other important crops [2]. *R. solanacearum* colonizes the root surface, attacks the plant through the xylem vessel, degrades the cell wall by releasing the enzymes cellulase and pectinase, and then inhibits nutrient and water translocation [3]. In severe attacks, *R. solanacearum* causes wilting, chlorosis, and death of tomato plants [4].

*Ralstonia solanacearum* can survive for a long period in the soil, which infects a broad plant species and wide geographical distribution, making pathogen prevention difficult. Bactericides used to control bacterial wilt are harmful to humans, food, and the environment. The use of biological control and resistant plants are the recommended means of reducing toxicity and residual effects [5]. The use of resistant varieties is considered less expensive and more environmentally friendly [6]. The resistance mechanism of tomato to bacterial wilt often involves biochemical defense mechanisms [7]. This mechanism includes the production of various biochemical compounds that have a negative effect on *R. solanacearum*.

Lowe-Power et al. [8] reported the involvement of R-genes in tomato plants resistant to bacterial wilt. R-genes induce the cell to produce a set of biochemical compounds that activate the immune system. These compounds prevent infection by facilitating metabolomic changes, including the production of primary and secondary metabolites related to defense systems [7]. These biochemical changes can be studied by metabolomics analysis. Metabolomics gives information about omics technology, revealing the total of primary and secondary metabolites in biological systems [9]. Zeiss et al. [7] used a metabolomics approach to identify the secondary metabolites in the tomato cultivars resulting from breeding programs with tomato plants infected by *R*. *solanacearum* using LC-MS. Nuclear magnetic resonance (NMR) based metabolomics has been applied successfully to determine the metabolites responsible for root-knot nematode resistance in tomatoes [10]. Bisht et al. [11] explained the advantage of using NMR spectroscopy to investigate tomato plant resistance to pests and diseases, as the technique is non-destructive, has high sensitivity and is highly reproducible. For these reasons, it is considered a suitable approach for detecting plant-biotic interactions [12].

A review study of Galeano Garcia et al. [13] stated several studies have reported on host-plant resistance to tomato plant diseases. Most of them, such as Zeiss et al. [7], used a reductionist approach by applying several analyses or technologies to detect metabolites and different plant cultivars. In this study, we applied NMR metabolomics analysis to revisit the chemical defense mechanisms of tomato to bacterial wilt caused by *R. solanacearum*.

## 2. Results

### 2.1. Disease Symptoms in Plants of Three Cultivars

**Figure 1:**
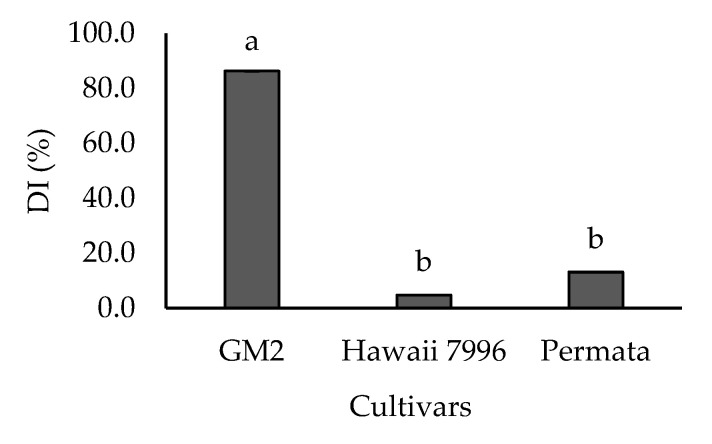
Disease Intensity (DI) of three cultivars tomatoes treated with *Ralstonia solanacearum.* Bars with the same letter were not significantly different at *p* = 0.05 based on Tukey’s HSD tests. Bars show the average disease intensity from 5 replicates.

Disease intensity scores showed that the three cultivars differed in their resistance levels to *R. solanacearum*, and two groups could be distinguished (Figure 1). The first group was resistant cultivars, including Permata and Hawaii 7996, with a DI value of 13.1% and 4.76%, respectively. In contrast, GM2 was categorized as a highly susceptible cultivar with a DI value of 86.31%.

### 2.2. Plant Metabolomics

**Figure 2:**
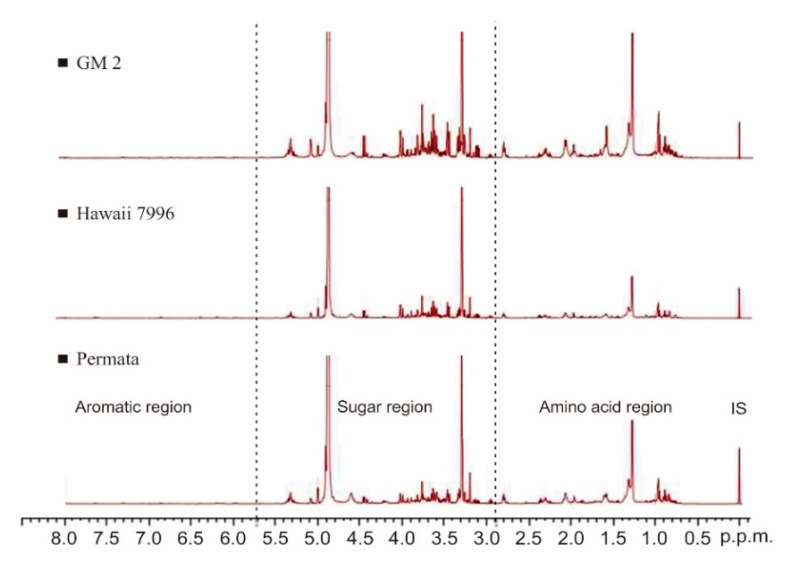
^1^H-NMR spectra of tomatoes show different peak intensities among three cultivars. IS: internal standard TMS.

The ^1^H-NMR spectra obtained from tomato leaf extract are shown in Figure 2. These spectra were processed by Mnova software using a semi-quantitative analysis and based on metabolite data from the references of previous studies [10]. Eleven primary metabolites were identified, including am ino acids, organic acids, and sugar groups (Table 1).

**Figure 3:**
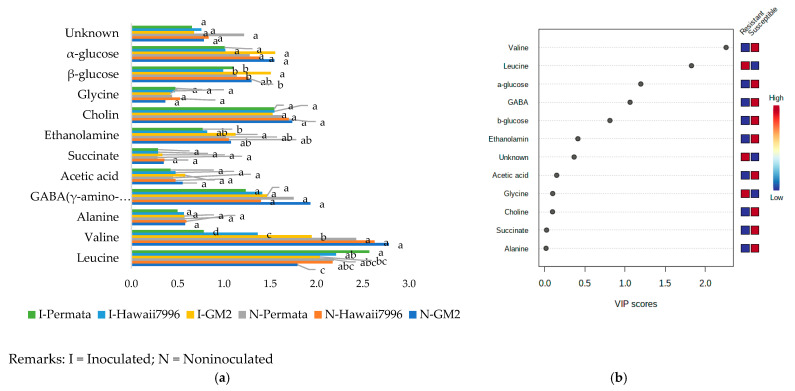
The concentration of peak assignment of tomato metabolites (**a**) and Variable Importance in Projection (VIP) of defined metabolites (**b**). Bars with the same letter in each group were not significantly different at *p* = 0.05 based on Tukey’s HSD tests.

The identified metabolites consisted of leucine, placed in the region of δ 0.94 with d, *J* = 0.7 Hz; valine (δ 1.00 [d, *J* = 7.0 Hz]) and (δ 1.05, [d, *J* = 7.0 Hz]; alanine (δ 1.45 [d, *J* = 7.2 Hz]); acetic acid (δ 1.95 [s]); γ-amino-butyric acid (GABA) (δ 1.88 [m], 2.37 [t, *J* = 7.2 Hz], and 2.96 [t, *J* = 7.08 Hz]); ethanolamine (δ 3.12, [t, *J* = 5.5 Hz]); Choline δ (3.19, [s]); Glycine (δ 3.5 [s]); β-glucose in the region δ 4.45 (d, [*J* = 7.8 Hz]); and α-glucose in the region δ 5.09 (d, [*J* = 3.76 Hz]) (Table 1). These metabolites varied in concentration between cultivars (Figure 3a). The Variable Importance in Projection (VIP) of defined metabolites shown in Figure 3b indicated that resistant cultivars had a higher concentration of leucine and glycine than susceptible ones. In contrast, susceptible cultivars had higher levels of valine, α-glucose, β-glucose, GABA (γ-amino-butyric acid), ethanolamine, acetic acid, choline, succinate, and alanine than resistant cultivars. The most promising metabolites with VIP values higher than 1.5 allowed identification of two important amino acids valine and leucine.

**Figure 4:**
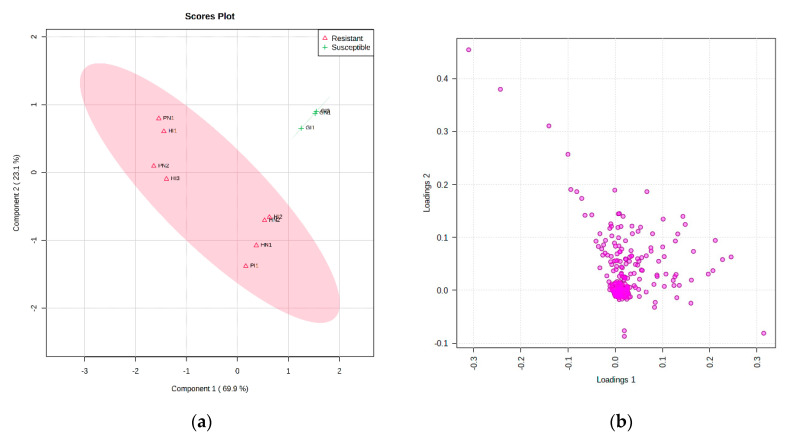
Scores (**a**) and loadings (**b**) of resistant and susceptible cultivars tomatoes with inoculation and non-inoculation. Non-inoculation of GM2 (GN), inoculation of GM2 (GI), Non-inoculation of Hawaii 7996 (HN), inoculation of Hawaii 7996 (HI), Non-inoculation of Permata (PN), inoculation of Permata (PI).

**Figure 5:**
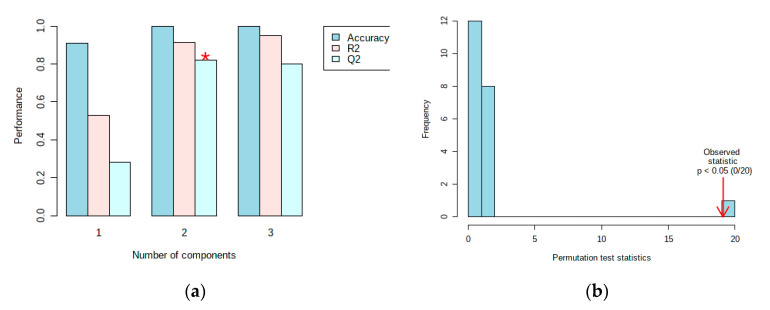
Validation of the PLS-DA model showing the variance of the response and predictive ability of the model (**a**) and a permutation test statistic (**b**). (*) indicates the optimal number of components included in order to make this model work.

The results of the ^1^H-NMR data analysis described different metabolites between resistant and susceptible cultivars in the scores plot (Figure 4a) and loading plot (Figure 4b). Before processing using multivariate data analysis, the data has been normalized with a feature-wise normalization i.e., auto scaling. This model explained 69.9% (PC1) and 23.1% (PC2) variation of the data and had a variance of the response R^2^ = 0.94 and a predictive ability Q^2^ = 0.88 (Figure 5a). The model was validated using a permutation test statistic, giving *p* < 0.05, as shown in Figure 5b. R^2^ is explained the calibration of model samples. However, Q^2^ described an estimate of the predictive ability of the model. Based on a study by Bevilacqua and Bro [14], when R^2^ and Q^2^ had a sufficiently small difference, the score plot models displayed a meaningful result. Therefore, it is clear that the result implied a good model.

**Figure 6:**
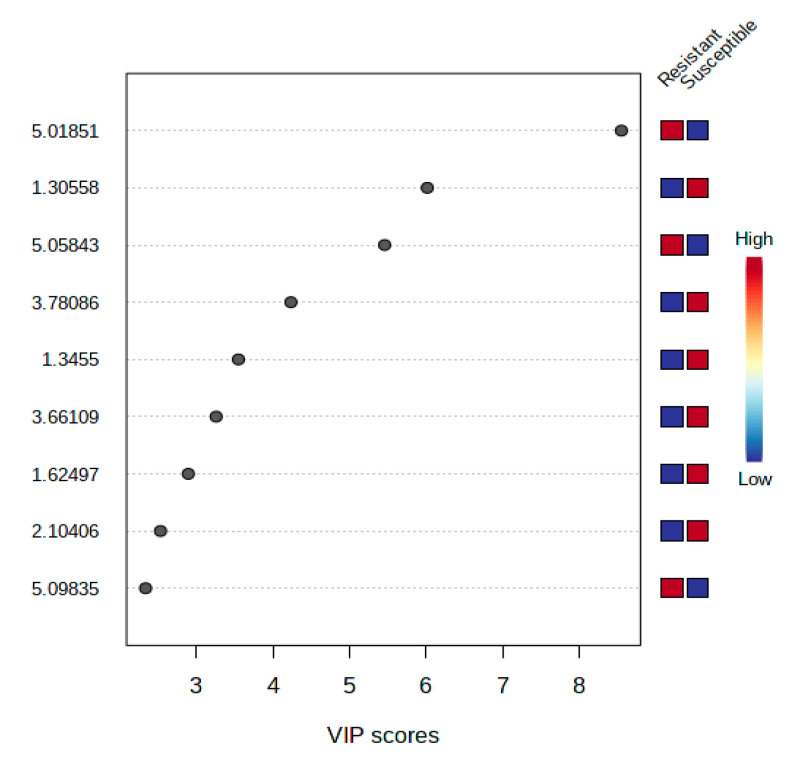
Variable Importance in Projection (VIP) of the metabolites in the top nine by the highest concentration.

The scores plot depicted perfect separation of resistant and susceptible cultivars, indicating that several metabolites play a role in the discrimination of these cultivars. However, some undefined metabolites that were also distinguishable between resistant and susceptible cultivars were presented clearly in the VIP (Figure 6). These metabolites were shortlisted among the top 9 with the highest concentration. VIP described that metabolites with chemical shifts at δ 5.02, δ 1.31, δ 5.06, δ 3.79, δ 1.35, δ 3.66, δ 1.62, δ 2.1, and δ 5.098 were sufficiently clear to differentiate between tomato resistant and susceptible to *R. solanacearum*. Moreover, resistant cultivars had higher levels of metabolites in the chemical shifts of δ 5.02, δ 5.06, and δ 5.098. In contrast, susceptible cultivars had higher levels of metabolites in the regions of δ 1.3, δ 3.78, δ 1.35, δ 3.67, δ 1.62, and δ 2.1.

**Figure 7:**
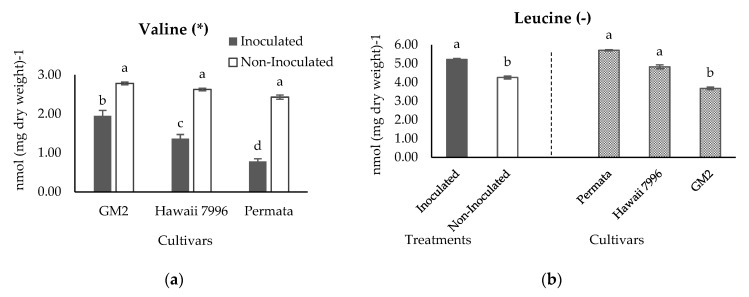
Concentration of Valine (**a**) and Leucine (**b**) in three cultivars tomatoes inoculated and non inoculated with *Ralstonia solanacearum*. Bars with the same letter in each group were not significantly different at *p* = 0.05 based on Tukey’s HSD tests. (*) indicates a significant interaction between treatments and cultivars; (**-**) indicates no significant interaction between treatments and cultivars; therefore, the histogram is separated between treatments and cultivars. Valine has the *p*_cultivars_ = < 0.0001, *p*_treatments_ = < 0.0001, *p*_cultivars × treatments_ = 0.0016. Leucine has the *p*_cultivars_ = 0.0005, *p*_treatments_ = 0.0075, *p*_cultivars × treatments_ = 0.1588.

Statistical analysis of 11 identified metabolites resulted that valine and leucine had a significant difference between resistant and susceptible cultivars based on Tukey’s HSD test with α = 5% (Figure 7). The histogram of valine presented in Figure 7a indicated that inoculation treatment decreased the valine concentration of resistant and susceptible cultivars. However, the fall of the valine level in the resistant cultivars (Hawaii 7996 and Permata) was higher than the susceptible cultivar (GM2). Valine concentration in the susceptible cultivars GM2 decreased 30 % from 2.78 nmol (mg dry weight)^−1^ to 1.95 nmol (mg dry weight)^−1^. Meanwhile, in the resistant cultivar (Hawaii 7996) decreased 48 % from 2.63 nmol (mg dry weight)^−1^ to 1.36 nmol (mg dry weight)^−1^. Furthermore, a resistant cultivar (Permata) decreased 68 % from 2.43 nmol (mg dry weight)^−1^ to 0.78 nmol (mg dry weight)^−1^.

The statistical analysis of leucine concentrations did not show an interaction between treatments and cultivars (Figure 7b). In consequence, leucine was evaluated separately between treatments and cultivars. The result revealed a rise in leucine after inoculation treatment. Meanwhile, Permata had a significantly higher level of leucine at 5.71 nmol (mg dry weight)^−1^, compared with the susceptible cultivar (GM2) at 3.68 nmol (mg dry weight)^−1^. A higher level of leucine was consistently observed in Hawaii 7996, a resistant cultivar, at 4.83 nmol (mg dry weight)^−1^. This result suggested that leucine is one of the metabolites that affected the distinction between resistant and susceptible cultivars. Permata, the most resistant cultivar, had a higher concentration of leucine, followed by Hawaii 7996, and the lowest was the susceptible cultivar GM2. Interestingly, leucine and valine showed a negative correlation at −0.68 analysed using Pearson correlation. This may underline that both amino acids are a member of the pyruvate family in terms of their biosynthesis. 

## 3. Discussion

Based on the disease intensity criteria, according to Aslam et al. [3], Hawaii 7996 and Permata are the most resistant plants against *R. solanacearum*. Based on a previous study by Truong and Wang [15], plants possess physical and chemical barriers, which allow them to mount a counterattack against pathogens such as fungi, nematodes, viruses, and bacteria. Resistant plants develop an immune system comprising several structural, biochemical, and protein-based defense systems to intercept pathogen invasion, including microbes, pests and herbivores [16]. Resistant plants involve highly complex biochemical defense mechanisms [17], including primary and secondary metabolites produced by inductive and defensive. Isah [18] explained that the defensive system in the plant had a relationship through the metabolite product, such as hypersensitive reaction, protein synthesis, and production of some phytoalexin.

Based on the identified metabolites successfully found in this study, several metabolites strongly differentiated between two resistant and one susceptible plants against *R*. *solanacearum*. These metabolites were identified as leucine, valine, and other unknown signals which placed in the region of 5.02, δ 1.31, δ 5.06, δ 3.79, δ 1.35, δ 3.66, δ 1.62, δ 2.1, and δ 5.098. Indeed, this study found that resistant cultivars had a higher concentration of leucine and metabolites in the chemical shifts of δ 5.02, δ 5.06, and δ 5.098. These metabolites strongly contributed to the separation between resistant and susceptible cultivars. This finding might indicate that leucine allegedly plays an important role in the tomato resistance to *R. solanacearum*. Indeed, leucine was reported to act as an essential element in antibacterial activity in the resistance mechanism [19]. In that study, leucine was demonstrated essential for antimicrobial activity by incorporated with a cationic charge into amino acid-based polymers. Mukherjee et al. [20] reported that the side-chain amino acid-based cationic polymers with pendant leucine moieties indicated efficient antibacterial activity.

Moreover, the role of leucine in conferring resistance to bacterial wilt was reported by Padnabhan et al. [21]. The study described that leucine-rich repeat (LRR) plays a role in the immune receptors. Leucine-rich repeat could recognize microbe-associated molecular pattern (MAMPs) and induce the plant immune response that inhibits pathogen access by various chemical and physical barriers. This in line with the study of Yuan et al. [22] and Chakraborty et al. [23]. The Leucine-rich repeat (LRR)-RLKs were identified to have a fundamental role in plant immunity and have function in signal transduction pathway upon the pathogen invasion. Thus, the leucine metabolic pathway constitutes an essential part of the plant immune system [24].

In addition, leucine is one of the amino acids that have a crucial role in the plant metabolism system, including contribution to the defense system. Fan et al. [25] also explained that many plant defensive compounds were derived from an amino acid, such as glucosinolates, a secondary metabolite produced by Brassicales to protect from a fungal pathogen, bacteria, and insect. Aliphatic glucosinolates are derived from alanine, leucine, valine, isoleucine, and methionine. Higher concentration of leucine upon infection with *Pseudomonas syringae* in *Arabidopsis thaliana* suggests the importance of this amino acid in the defense system mechanism in plants [26]. Hence, Zeiss et al. [7] also underlined the branched-chain amino acids (BCAA), including isoleucine, leucine, and valine, contribution to the pre-existing secondary metabolite for the plant defense system. The secondary metabolites associated with host-plant resistance are influenced by amino acids that modulate to cross-talk of jasmonic acid (JA) and salicylic acid (SA). JA and SA are known as secondary metabolites-derived signals that regulate plant stress responses [27].

Besides leucine, another amino acid that contributes to the separation between resistant and susceptible cultivars was valine. This study revealed that inoculation with *R. solanacearum* lowered valine concentration in both resistant and susceptible cultivars. Valine is one of the amino acids that isare important for the plant metabolism system. Mikkelsen and Halkier [28] explained that numbers of amino acids included valine, alanine, leucine, isoleucine, methionine, phenylalanine, tyrosine, and tryptophan generate glucosinolates which had great potential for improving resistance against herbivores and other pathogens. Furthermore, as part of the BCAA, both valine and leucine share a similar biosynthesis pathway, the pyruvate-family amino acid [29]. Thus, it may explain the negative correlation between valine and leucine. When the plant synthesis a high concentration of leucine, then a lower concentration of valine may occur as a consequence of having similar precursors.

Another interesting point is that several metabolites (a group of undefined metabolites) also contributed to the separation between resistant and susceptible varieties. Furthermore, according to Al Sinani and Eltayeb [30], generally, plants have the ability to involve and adapting several metabolites for improving a wide range of resistance mechanisms toward pathogens. For instance, plants synthesized and developed various biochemical compounds to counterattack the infection of the pathogen. Many studies had been reported the secondary metabolites associated with *R. solanacearum*. These metabolites vary in each genotype and are based on the method used to detect metabolomic in that plant. Zeiss et al. [7] studied tomato-host-plant resistance to *R. solanacearum*. In that study, they observed that flavonoids, HBAs, and HCAs had important roles in the defense mechanism of tomato against *R. solanacearum*.

## 4. Materials and Methods

### 4.1. Bacterial Inoculum

This research used bacterial wilt (*R. solanacearum*), race 1 and biovar 3, which was obtained from the culture collection of Plant Protection Laboratory, Faculty of Agriculture, Universitas Gadjah Mada, Yogyakarta, Indonesia. It had been collected from wilted tomato plants from Seyegan sub-district, Sleman district, Yogyakarta, Indonesia. Firstly, infected tomato plants were cleaned with distilled water, the stem cut into 0.5 cm and sterilized with alcohol 70%, followed by soaking into sterile water for 10 min. The bacterial suspension was streaked into YPGA (yeast peptone glucose agar) media containing yeast extract (5 g), 10 g peptone and glucose, 15 g agar in 1 L distilled water and then incubated for 48 h. Furthermore, the bacteria cells were suspended in sterile distilled water, and the density of inoculum was set to 1 × 10^8^ cfu·mL^−1^ [31].

### 4.2. Plant Materials

Three tomato cultivars, GM2, Permata, and Hawaii 7998 were used in this study. GM2 is a cultivar owned by the Faculty of Agriculture, Universitas Gadjah Mada. It was used as the susceptible cultivar, as it is known to have a high susceptibility to *R. solanacearum*, as reported by Maulida et al. [32]. Permata is a commercial tomato hybrid produced by a private company, East West Seed Indonesia, Purwakarta, West Java, Indonesia. Based on the cultivar description, this hybrid is resistant to bacterial wilt caused by *R. solanacerum* [33]. Hawaii 7996 is a cultivar introduced from World Vegetable Center (WVC), previously identified as resistant to *R. solanacearum* [34].

### 4.3. Experimental Procedure

The study was carried out in a greenhouse, Faculty of Agriculture, Universitas Gadjah Mada. Tomato seeds were sown in the sterile media added nitrogen, phosphorus, and potassium (NPK) fertilizer (1:1:1) with a ratio of 1:50 (*w*/*w*) (fertilizer and sterile soil). Twenty-one days after germination, these plants were moved into polybag 130 × 130 mm individually. Seven days after transplanting, tomato plants were inoculated with bacterial wilt (100 mL per plant). Before infection, tomato roots were injured by the knife through plant media and then 100 mL of inoculum solution with a density of 1 × 10^8^ cfu·mL^−1^ was poured into the wounded area to make infection through the plant.

Observation and scoring of the disease symptoms were based on Chen et al. [35]; 1 = no symptoms, 2 = Less than a half of leaves wilted, 3 = a half of leaves being wilted, 4 = All of leaves wilted, 5 = whole the plant wilted and dead. Furthermore, the disease intensity was used as a classification of plant resistance. Cultivars have a disease intensity of 0–20% were categorized as high resistant (HR), 21–30 was Resistant, 31–40% was Moderately Resistant (MR), 41–50 was Moderately Susceptible (MS), 51–60 was Susceptible (S), 61–90 was Highly Susceptible (HS), and 91–100 was Extremely Susceptible (ES) [6]. Hence, DI was calculated using the formula of Chen et al. [35]:

$$DI=\frac{\sum n}{5N}\times 100\%$$

Remarks: ∑n  = A sum from ratings of all plants scored at 1–2, e.g., ∑ (1A+2B+3C + 4D + 5E), whereas A, B, C, D, and E were a total of the plants that categorized at score 1, 2, 3, 4, and 5; N = The number of inoculated plants; 5 = the highest category of symptom scale.

The leaves of plants were harvested 14 days after *R. solanacearum* inoculation. Leaves were collected 5 g per plant for freez drying process. The leaves were ground with mortar and pestle in liquid nitrogen then immediately sent to freeze drier for 48 h and then taken about 50 mg for NMR analysis The method used in this study was based on the modification of Schripsema and Dagnino [36].

### 4.4. Statistical Analysis

This study was arranged in a Split Plot Design with the cultivars served as the main factor (GM2 vs. Permata vs. Hawaii 7998) and the bacterial infection as a sub-factor (Healthy vs. infected). The collected data (Disease intensity and the semi-quantitative concentration of identified metabolites) were statistically analysed by analysis of variance (ANOVA) and continuous with Tukey’s HSD test for determining the significant difference with α = 5%. Furthermore, the multivariate data were performed using Partial Least-Squares Discriminant Analysis (PLS-DA) by MetaboAnalyst Software version 5.0 (www.metaboanalyst.ca accessed on 27 May 2021).

### 4.5. ^1^H-NMR Measurement

NMR conditions were based on López-Gresa et al. [37] modified by Afifah et al. [10]. Approximately 50 mg freeze-dried tomato leaves were used for ^1^H-NMR analysis. Each of the samples was placed into a 2 mL Eppendorf tube, added 1 mL of deuterated methanol (CD_3_OD) containing 0.05% of internal standard (trimethyl silyl-3-propionic acid (C_6_H_14_O_2_Si)). The tubes were vortexed for 1 min and sonicated for 20 min. Subsequently, the tubes were centrifuged at 13,000 rpm for 10 min. Approximately ~800 μL of the supernatant was transferred into the NMR tube for ^1^H-NMR measurement using a 500 MHz ECZR-JEOL NMR spectrometer (JEOL USA Inc., Peabody, MA, USA). Each NMR spectra was recorded at 30 °C, 128 scans, 26 s acquisition time, 0.16 Hz per point, with pulse’ width of 30 and relaxation delay of 1.5 s.

### 4.6. Data Quantification

Spectra from ^1^H-NMR were analysed using Mnova software version 11 from Mestrelab Research (Timber Glen, Escondido, CA, USA) for metabolites identification and semi-quantitative analysis following the published data of Kim et al. [38], López-Gresa et al. [37], and Escudero et al. [39]. Previously, the spectra were manually corrected, phased, and baselined correction. Furthermore, an internal standard (trimethyl silyl-3-propionic acid (C_6_H_14_O_2_Si)) was calibrated by setting at 0.0 ppm. Residual water in a range of δ 4.7–4.9 and methanol in a range of δ 3.28–3.34 was excluded from the analysis.

## 5. Conclusions

^1^H-NMR is the appropriate metabolomic study to reveal the biochemical compounds that associated to the defense mechanism in the tomato plants against *R. solanacearum*. This study identified that leucine, valine, and other metabolites which placed in the region of 5.02, δ 1.31, δ 5.06, δ 3.79, δ 1.35, δ 3.66, δ 1.62, δ 2.1, and δ 5.098 were alleged to differentiate between resistant and susceptible tomato against *R. solanacearum*. Leucine dominated in the resistant plant (Permata). Meanwhile, resistant cultivars had a significant decrease of valine after inoculation about 1.5–2 times compared to the susceptible cultivar. Leucine is alleged to contribute to the immune system and the existence of secondary metabolites for defense systems in plants toward bacterial wilt.

## Figures and Tables

**Table 1:** Peak assignments for the ^1^H-NMR spectrum of tomato leaf in Methanol-d4 (MeOD_4_).

Metabolite	Chemical Shift (ppm) and Coupling Constants (Hz)
Leucine	0.94 (d, *J* = 0.7 Hz)
Valine	1.00 (d, *J* = 7.0 Hz); 1.05 (d, *J* = 7.0 Hz)
Alanine	1.44 (d, *J* = 7.2 Hz)
Acetic acid	1.94 (s)
Succinate	2.53 (s)
GABA (γ-amino-butyric acid)	1.88 (m); 2.37 (t, *J* = 7.2 Hz); 2.95 (t, *J* = 7.08 Hz)
Ethanolamine	3.12 (t, *J* = 5.5 Hz)
Choline	3.19 (s)
Glycine	3.5 (s)
β-glucose	4.45 (d, *J* = 7.8 Hz)
α-glucose	5.09 (d, *J* = 3.76 Hz)

Abbreviations: d = doublet; m = complex multiplet; s = singlet; t = triplet; *J* = the coupling constant.

## Data Availability

The data presented in this study are available within the article.
